# Coupling between Catalytic Loop Motions and Enzyme Global Dynamics

**DOI:** 10.1371/journal.pcbi.1002705

**Published:** 2012-09-27

**Authors:** Zeynep Kurkcuoglu, Ahmet Bakan, Duygu Kocaman, Ivet Bahar, Pemra Doruker

**Affiliations:** 1 Department of Chemical Engineering and Polymer Research Center, Bogazici University, Bebek, Istanbul, Turkey; 2 Department of Computational and Systems Biology, and Clinical & Translational Science Institute, School of Medicine, University of Pittsburgh, Pittsburgh, Pennsylvania, United States of America; UNC Charlotte, United States of America

## Abstract

Catalytic loop motions facilitate substrate recognition and binding in many enzymes. While these motions appear to be highly flexible, their functional significance suggests that structure-encoded preferences may play a role in selecting particular mechanisms of motions. We performed an extensive study on a set of enzymes to assess whether the collective/global dynamics, as predicted by elastic network models (ENMs), facilitates or even defines the local motions undergone by functional loops. Our dataset includes a total of 117 crystal structures for ten enzymes of different sizes and oligomerization states. Each enzyme contains a specific functional/catalytic loop (10–21 residues long) that closes over the active site during catalysis. Principal component analysis (PCA) of the available crystal structures (including apo and ligand-bound forms) for each enzyme revealed the dominant conformational changes taking place in these loops upon substrate binding. These experimentally observed loop reconfigurations are shown to be predominantly driven by energetically favored modes of motion intrinsically accessible to the enzyme in the absence of its substrate. The analysis suggests that robust global modes cooperatively defined by the overall enzyme architecture also entail local components that assist in suitable opening/closure of the catalytic loop over the active site.

## Author Summary

Protein loops have critical roles in ligand binding and catalysis. An unresolved issue in this context is the extent to which the intrinsic dynamics of proteins predispose loops to perform their molecular function. In this work, we (i) critically examine the structural changes undergone by functional/catalytic loops based on a set of enzyme crystal structures in the presence/absence of a ligand, and (ii) examine to what extent those motions are correlated with, or driven by, the global modes that are predictable using simplified, physics-based models. Using a dataset of 117 structures for ten enzymes of different sizes and oligomerization states, we show that the collective modes defined by the protein topology favor loop rearrangements in reasonable agreement with those experimentally observed upon activation. These results suggest that simple but robust motions encoded by the entire architecture, not the local binding site only, assist in binding of the ligand, positioning of the catalytic loop, and/or sequestration of the catalytic site, which in turn, enable efficient catalysis.

## Introduction

An issue yet to be resolved is the extent to which the intrinsic dynamics of proteins predispose them to ligand binding. Is there any correlation between local functional events such as loop rearrangements involved in ligand binding and the collective motions intrinsically accessible to the protein prior to ligand binding? To what extent do the structure-encoded global modes of motions (e.g. domain opening/closing, exposure or burial of active sites, cooperative conformational switches in allosteric proteins) simultaneously engage loop motions that facilitate functional interactions? Or, are loop reconfigurations mainly induced on a local scale by the ligand?

Notably, two different views have been advanced in recent years in linking protein dynamics and function: (i) enzyme structural flexibility affects its catalytic reactivity [1]–[4], (ii) catalysis is independent of collective dynamics [5]–[7]. The second view is supported by the limited mobility of catalytic residues in the collective motions of the protein (due to the requirement of precise positioning for chemical reactivity). Recent studies show that the preorganization of the active site is a rate-limiting factor in catalysis, while conformational dynamics help reorganize structural elements near the catalytic site [8].

The global motions of enzymes, also called slowest or softest due to their low frequency or small effective force constants, have been shown in numerous studies [9], [10] to be robustly defined by the evolutionarily selected fold. It is conceivable that these structure-encoded modes play a role in facilitating the enzymatic activity, for example, by favoring structural changes that enable efficient recognition and binding of the substrate/ligand [11]. There is experimental evidence that the loss of conformational motion affects the enzymatic mechanism, even though the structure and electrostatics are preserved [4], while recent work showed that electrostatic preorganization, not conformational motions, makes the largest contribution to catalysis [7]. Our examination of triosephosphate isomerase (TIM) collective dynamics suggests that there is a coupling between the global dynamics of the molecule and the local motions of the catalytically active loop 6 [12], [13]. As illustrated in the Supporting Information (SI) Figure S1, the experimentally observed closure of loop 6 over the ligand is in accord with the essential/principal mode of motion observed in molecular dynamics (MD) simulations of TIM; furthermore this first principal mode extracted from MD by essential dynamics analysis (EDA) [14] is in agreement with the global (softest, lowest frequency) mode predicted for the dimer using the anisotropic network model (ANM) [15], [16]. Collective monomeric counter-rotations, which are not evident in experimental data, appear to be coupled to the functional loop's opening/closure over the active site. Moreover, experiments for TIM indicate that loop closure is not ligand-gated and emerges as an intrinsic motion of the apo enzyme [17]. While these observations signal a role of global dynamics in facilitating functional loop motions, there has been no systematic study of enzyme dynamics in relation to loop motions to establish the generality of these observations, apart from a recent study by Jernigan and coworkers where attention has been invited to the importance of slow modes for functional loop motions [18].

With the rapid accumulation of both apo and liganded structures (usually open and closed forms, respectively) for a given protein in the Protein Data Bank (PDB) [19], and with the development of analytical models and tools for rapid estimation of intrinsic dynamics, we are now in a position to (i) critically examine the structural changes undergone in recognition loops and/or catalytic sites based on structurally resolved proteins in the presence/absence of a ligand and (ii) examine to what extent those motions are correlated with, or driven by, the global modes that are predictable using simplified, physics-based models.

To this aim, we focus on a series of enzymes, where loop motions relevant to function have been experimentally detected (Table 1). In each case, we have a set of structures containing the apo and ligand-bound forms, which differ particularly in their loop regions. As listed in Table 1, the root-mean-square deviation (RMSD) between the open and closed forms varies in the range 0.9–3.9 Å (after optimal superposition of the open and closed structures to eliminate rigid-body translations and rotations), while the loop RMSD varies between 3.5 and 14.5 Å; and the tip residues of the loops are displaced by 6.7 to 25.0 Å between the open and closed conformations. On the other hand, the internal RMSDs of the loops, obtained after structural alignment of the isolated loops, are lower than 5.5 Å (Table 1), suggesting that the large displacements of the loops on the proteins are to a large extent due to the rigid-body displacements, which may be coupled to the collective motions of the enzymes. Notably, four out of ten enzymes (TIM, protein tyrosine phosphatase (PTP), L-lactate dehydrogenase and 3-dehydroquinase) exhibit almost a rigid-lid type closure with a loop internal RMSD less than 2 Å (Table 1).

**Table 1:** Dataset of enzymes with functional loops that close over the active site.

			RMSD (Å)			
Enzyme	Number of residues^a^	Loop residues	Overall protein^b^	Loop^c^	Isolated loop^d^	Loop tip motion (Å)
Protein Tyr phosphatase (PTP)	305	352–361	0.9	3.5	1.0	7.0
H*ha*I methyltransferase	327	80–100	3.9	14.5	5.3	25.0
OMP decarboxylase	267 (x2)	203–218	2.7	9.6	4.0	14.7
β 1,4-galactosyltransferase	288	345–365	3.4	11.6	5.5	21.5
L-lactate dehydrogenase	317 (x4)	81–91	0.9	4.3	1.0	6.7
3-dehydroquinase	252 (x2)	227–239	1.2	5.1	1.6	10.1
Biphosphate aldoase	307 (x2)	176–191	1.8	7.2	3.3	16.5
Triosephosphate isomerase (TIM)	248 (x2)	166–176	1.0	4.5	1.1	7.9
Enolase	436 (x2)	34–50	0.9	3.7	2.8	8.8
Pyruvate mutase	295 (x4)	118–134	2.4	9.7	4.2	18.0

a Oligomerization state (number of monomers) is specified in parentheses.

b RMSD between the representative open (apo) and closed (liganded) structures listed in Table S1 in Text S1, based on C*^α^* atoms.

c RMSD of the loop region calculated after aligning the same apo and bound structures using the C*^α^* atoms. The value is reported for the loop in chain A for multimers.

d RMSD obtained after superimposition of the loop region only.

The approach we undertake is the following: (i) to determine the dominant conformational changes of the functional loops by performing a principal component analysis (PCA) of the available crystal structures for each enzyme as well as by direct examination of two structures representatives of the open and closed forms (see Table S1 in Text S1), (ii) to determine collective modes of motion of a representative unliganded member using the ANM, and (iii) to examine the overlap between functional loop reconfiguration derived from experimental data and structure-based motions predicted by the ANM, as explained in previous work [11]. Additionally, we will extract essential motions from MD simulations for PTP and TIM as two case studies to further establish the correlation, if any, between computationally predicted loop motions, and those experimentally observed. We will show that a few well-defined, energetically accessible collective modes of motions encoded by the entire architecture, not by the local binding site only, favor suitable repositioning of the catalytic loop, which in turn, enable the predisposition of the active site to catalytic activity.

## Results

### Overview of dataset, method of approach, and results

Calculations were performed for a dataset of 117 structures from the PDB corresponding to 10 enzymes (Tables 1 and S1), with 2 to 28 structures resolved in different forms per enzyme. Among them, *Hha*I methyltranferase (M. *Hha*I) is a DNA-binding enzyme; and all others bind ligands of various sizes. They contain *s* = 10–21 residue long loops that close over the active site during reaction. By this means, a catalytic residue located on the loop is correctly positioned in the active site and the site is protected from solvent during catalysis.

We compare two sets of data generated for each enzyme: *experimental*, derived from the structures known for the enzyme; and *computational*, predicted for a representative unliganded structure (indicated as open structure in Table S1 in Text S1). Of interest is to assess the correspondence, if any, between the experimentally observed (local) loop motions, and the predicted loop motion as driven by the soft (global) ANM modes. As a metric, we use the overlap *O_1j_*≡|***p*** *^(1)^*. ***u*** *^(j)^*| between the dominant motion inferred from experiments (expressed by *3N*-dimensional unit directional vector, ***p*** *^(1)^*, also called PC1 if obtained by PCA or deformation vector if calculated from the difference between open and closed forms; see Methods) and the *j^th^* eigenmode ***u*** *^(j)^* predicted by the ANM. *O_1j_* varies by definition in the range [0, 1]. An overlap close to 1 means that the experimentally observed structural change is essentially driven by the mode *j*. Another metric is the *cumulative overlap*, a summation over a subset of p modes (see Methods), describing the fractional contribution of *p* modes to the (experimentally) observed deformation.

Figure S2 displays the *O_1j_* values for the slowest 40 modes (bars) and their *cumulative overlap* (curve) for each enzyme. In six out of ten enzymes, there is at least one mode with an *O_1j_*>0.4, and a cumulative overlap of 0.7 or higher is attained in 7/10 cases, suggesting that the soft modes facilitate, if not enable, functional loop motions.

We further made a direct assessment of the orientational correlation between the loop motions observed in experiments and those predicted by computations. To this aim, we evaluated the correlation cosine 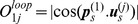, between the *3s*-dimensional subvectors ***p***_s_^(*1*)^ and ***u***_s_^(*j*)^ corresponding to the loop regions of ***p*** *^(1)^*and ***u*** *^(j)^*. O*_1j_^loop^* will be shortly called *loop overlap*. Table 2 shows that a loop overlap of 0.57≤O*_1j_^loop^*≤0.86 is achieved by at least one mode (among the softest 10; written in parentheses) in each examined enzyme (*column 2*). *Column 3* lists the softest mode that yields a loop overlap higher than 0.5; and *column 4*, the modes, among the softest 10, that yield a loop overlap of 0.5 or higher.

**Table 2:** Comparison of experimentally observed and theoretically predicted loop motions.

	Normalized loop overlap (orientational)			Enhancement factor for weighted-average loop overlap^d^
Enzyme	Highest overlap^a^	Slowest mode with^b^ *O≥0.5*	Slow modes with^c^ *O≥0.5*	<*O*|_1–10_>
PTP	0.72 (6)	0.51 (2)	2, 6	1.8
H*ha*I methyltransferase	0.82 (1)	0.82 (1)	1, 4, 7, 9–10	5.2
OMP decarboxylase	0.79 (7)	0.72 (1)	1, 6–9	11.6
β 1,4-galactosyltransferase	0.63 (4)	0.55 (1)	1, 4	7.1
L-lactate dehydrogenase	0.74 (1)	0.74 (1)	1–4, 7–9	16.1
3-dehydroquinase	0.76 (10)	0.65 (5)	5–7, 10	2.5
Biphosphate aldoase	0.61 (2)	0.61 (2)	2, 6, 8–9	3.7
TIM	0.78 (10)	0.64 (4)	4, 7, 10	3.8
Enolase	0.57 (5)	0.54 (3)	3–5	2.6
Pyruvate mutase	0.69 (8)	0.69 (8)	8	5.2

a, b, c Overlap between loop reconfiguration observed in experimental structures and the structural change predicted for the loop by the ANM.

a Highest overlap achieved by a single mode and corresponding mode number in parentheses.

b Slowest ANM mode (in parentheses) that shows an overlap greater than 0.5, and the corresponding overlap.

c Slow modes that show overlap greater or equal to 0.5.

d The ratio <O|*_loop_*>*_p_^ANM^*/<O|*_loop_*>*_p_^random^* (see Eq. 1 and main text).

We also calculated the *weighted-average overlaps*, <O|*_s_*>*_p_*, averaged over *p* = 10 modes (see Eq. 1 in Methods) evaluated for segments of *s* consecutive residues. Figure S3 displays <O|*_s_*>*_p_* for the catalytic loop (*s*-residue long), calculated for successive sets of 10 modes (shifting windows along the abscissa of 3*N*-6 ANM modes). A general trend of decreasing loop overlap with increasing mode number is observed for all enzymes.

As a further test, we compared weighted-average loop overlaps, calculated for the softest 10 modes, to those accomplished by randomly generated modes. To that aim, we evaluated the *difference* Δ<O|*_s_*>*_p_* = <O|*_s_*>*_p_^ANM^*−<O|*_s_*>*_p_^random^* at the loop region of *s* residues, and repeated the calculations for all successive windows of *s* residues along the protein sequence. The goal was to test whether the resulting ‘difference profiles’ as a function of residue (sliding window) index would distinguish the loop regions as regions of high overlap with ANM softest modes (e.g. *p* = 10 of them). The difference profiles presented in Figure S4 clearly indicate that for the most part the catalytic loop regions (the positions of which along the sequences are indicated by red stars and dashed vertical lines) are distinguished by their high overlap with slow modes, in support of the correlation between structure-encoded soft modes and functional loop reconfigurations. The last column in Table 2 shows that the *enhancement factor* calculated as the ratio <O|*_s_*_ = *loop*_>*_p_^ANM^*/<O|*_s = loop_*>*_p_^random^*. Notably, the enhancement factor varies between 1.8–16.1, with PTP exhibiting the smallest enhancement, and L-lactate dehydrogenase, the largest.

In summary, in each studied protein, at least one of the top-ranking (energetically favorable) 10 collective modes predicted by the ANM yields a high loop overlap, and the weighted-average overlap achieved by these soft modes at the loop region is enhanced by a factor of 6.0 on average (over 10 proteins) compared to randomly generated modes. These data further support the view that the seemingly ‘local’ loop reconfigurations inferred from experimental data are not decoupled from the global modes intrinsically encoded by the overall structure. On the contrary, global modes generally exhibit higher overlaps with the functional loop motions than local (high frequency) modes (Figure S3) or random modes (Figure S4).

Below we describe in more details the results for four enzymes.

### Protein Tyrosine Phosphatase (PTP)

**Figure 1:**
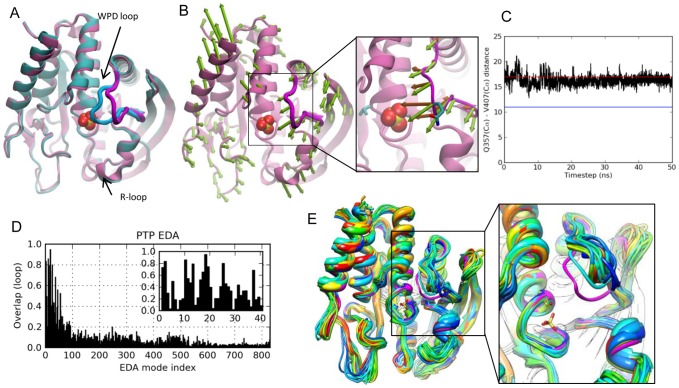
Results for PTP. **A.** *Yersinia* PTP crystal structures with open/apo (*mauve* backbone and *magenta* loop; PDB id 1YPT [22]) and closed/sulfate-bound (*cyan*/*blue* loop; PDB id 1YTS [21]) conformations of WPD loop are shown.. **B.** ANM mode 2, the slowest mode that yields an overlap higher than 0.5 with the experimentally reconfiguration at the loop region. The close-up view compares computed (ANM2) motions (*green* arrows) and the structural change observed between resolved apo and liganded structures (*dark orange* arrows). Side-chain atoms of Gln357 (on the loop) and Val407 (at the active site) are shown in stick representation. **C.** Change in the distance (Å) between Gln357 and Val407 C^α^ atoms in the MD run, compared to the distances for closed (*blue* line) and open (*red* line) structures. **D.** Loop overlap between essential modes and experimentally observed reconfiguration. **E.** MD snapshots from every 2.5 ns, compared to the closed form (*magenta*) and the open (initial) form (*dark blue*), with the inset showing a slightly rotated, enlarged view.

Protein tyrosine phosphatases form a superfamily of enzymes that regulate the tyrosine phosphorylation levels in signal transduction pathways together with the action of protein tyrosine kinases. Specifically, PTPs catalyze the hydrolysis of phosphate moiety in phosphotyrosine-containing proteins. Class I cytoplasmic PTPs include human PTP (PTP1B) and *Yersinia* PTP (YopH), which show low sequence identity (∼20%) [20]–[22], but share a structurally conserved catalytic domain of ∼280 residues [21], namely an eight-stranded mixed β-sheet wrapped by seven α-helices (Figure 1A). One important feature of PTPs is the WPD loop, which carries the conserved catalytic residue Asp356 (in YopH) and closes over the active site upon binding of the substrate (open and closed conformations of the loop are shown in Figure 1A). Similar to TIM, loop closure correctly positions the functional residues around the ligand and shields the site from bulk solvent during catalysis [21]. Standard and targeted MD simulations [23], [24] on PTP1B have identified important regions (S-loop, R-loop) that are possibly related to ligand binding and closure of the WPD loop, respectively (Figure 1A).

#### PCA results from experiments

Our ensemble consists of 16 PTP crystal structures (Table S1 in Text S1). The first PCA mode, which corresponds to 53% of the total variance (Table S2 in Text S1), describes mainly the variation of the WPD loop between open and closed conformations. Interestingly, the other solvent-exposed loops and helices do not exhibit any motion in the principal modes, consistent with the conformations shown in Figure 1A. Experiments for PTP have indicated that loop closure is not ligand-gated and emerges as an intrinsic motion of the apo enzyme [25]. So our question here is whether the WPD loop closure described by PC1 is coupled to the collective motions of PTP.

#### Overlap with ANM predictions

**Figure 2:**
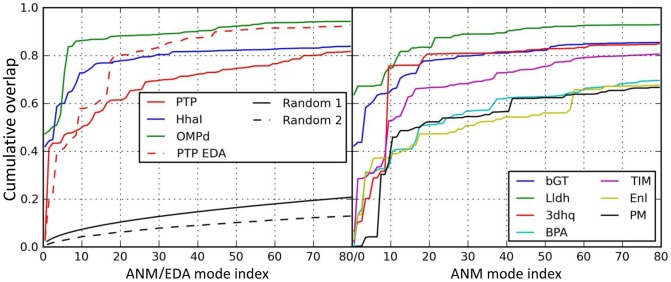
Cumulative overlaps between computationally predicted and experimentally observed structural changes. Results are shown for all studied enzymes as labeled. Computational results refer to ANM modes (for all) and EDA modes for PTP. Random 1 curve shows the cumulative overlap that would be obtained by randomly generated normal modes. Random 2 shows the average of cumulative overlap curves for randomly selected high frequency modes.

The overlap between experimental data (PC1) and the ANM modes is calculated to detect any correlation between loop closure and the slow modes. We performed ANM calculations for open structure with PDB identifier 1YPT [22]. ANM mode 2 (ANM2) yielded highest overall overlap (0.41, Figure S2) with PC1. We depict fluctuations along ANM2 using arrows (*green*) pointing in the loop closure direction in Figure 1B. In the close-up view, ANM2 arrows are compared to the loop closure observed in crystal structures (*dark orange arrows*). As to the WPD loop overlap, two out of 10 slowest modes exhibit correlation cosines above 0.5 (0.72 for mode 6, and 0.51 for mode 2; see Table 2). Further, slowest 10 ANM modes yield an overlap enhancement of 1.8 relative to random modes, which is actually the lowest enhancement factor in the dataset. Finally, cumulative overlap (see Methods) plots show that 20 modes (2.4% of all modes) explain 60% of the structural variance. (Figures 2A and S2). Note that slow modes 2 and 6 alone account for 45% of the variance.

#### MD results

We also performed a 50 ns MD run of the apo enzyme. Similar to ANM results, the highest correlation with PCA1 achieved by individual EDA modes is 0.44. However, the loop overlap values achieved by the essential modes at the low frequency region of the spectrum are considerably higher than those at the intermediate and high frequency ranges, as illustrated in Figure 1D. These results are in accord with previous 10 ns MD simulations on YopH [26]. Essential modes consistently show WPD loop's half-closure in apo form. In Figure 1C, the time evolution of the distance between active site residue Val407 and loop residue Gln357 is plotted, showing that the loop has a tendency to occasionally move towards the closed conformation (indicated by the blue horizontal bar), although it never reaches the closed form. Note that these runs are performed for the unliganded PTP so as to assess the conformational dynamics of the enzyme in the absence of ligand binding. The ‘incomplete’ closure of the loop in simulations may thus result from the lack of interactions, (mainly electrostatic, between the charged residues on the loop and the ligand) which would otherwise stabilize the closed state. In Figure 1E, snapshots from every 2.5 ns are shown to display halfway closed loop configurations from the simulation. Overall, an intrinsic feature of PTP functional dynamics is the flexibility of WPD loop to fluctuate in the functional direction, confirmed by both ANM and MD results.

### *Hha*I methyltransferase (M.*Hha*I)

**Figure 3:**
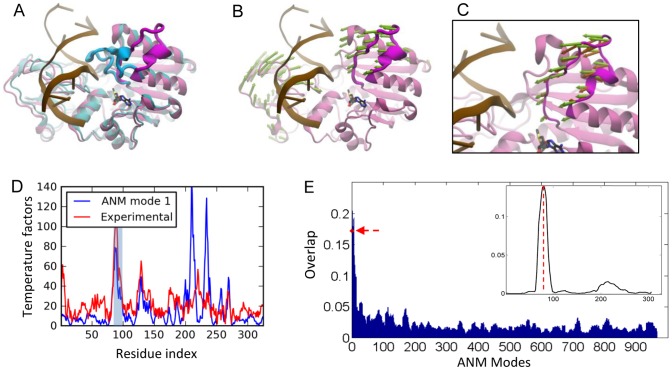
Results for M.*Hha*I methyltransferase. **A.** Open (*mauve/magenta* loop; PDB id 2HMY [29]) and closed (*cyan/blue*; PDB id 3HMT [48]) forms of M.*Hha*I are shown with bound DNA (*light brown*). **B.** ANM mode 1 direction is shown by *green* arrows. **C.** A close-up view of ANM1-predicted loop motions compared to experimental changes (*orange* arrows). **D.** Theoretical temperature factors calculated using ANM mode 1 (blue curve) compared to experimental B-factors (red curve). Loop region is highlighted in *light blue*. **E.** Weighted-average loop overlap, (<O|*_s_*>*_p_* averaged over *p* = 10 modes, evaluated for the loop region of *s* residues, repeated for successive sets of 10 modes (shifting windows along the abscissa). The bars show the significantly higher overlap with experimentally observed loop reconfiguration achieved by the softer ANM modes, and there is a sharp decrease in overlap with increasing mode numbers. The red arrow along the ordinate indicates the overlap achieved by the first 10 modes. The inset plots the weighted-average overlaps <O|*_s_*>*_p_* (based on *p* = 10 softest modes) relative to that of random modes, repeated for successive windows of *s* residues along M*Hha*I sequence. The vertical dashed line indicates the sequence position of the functional loop.

M.*Hha*I catalyzes the methylation of cytosine residues located in specific DNA sequences with the aid of a cofactor (S-adenosyl-L-homocysteine). M.*Hha*I is a monomeric enzyme, which positions the DNA between its large (Rossmann fold) and small domains [27] (Figure 3A). The catalytic nucleophile Cys81 is located on a long, flexible loop, with tip residue displaced by 25 Å when it binds the DNA [27]. This is accompanied by a flip of the target cytosine out of the DNA helix into the active site. The collective motions of the M.*Hha*I have been proposed to facilitate the base flipping observed in the ternary complex in a study using elastic network model [28].

#### PCA results

We analyzed an ensemble of 29 *Hha*I structures - two unbound, and the rest, ligand-bound. The closure of the loop (movement along PC1) explained 90% of the structural variability in the dataset and 8% of the remaining variability was attributed to the structural changes observed in the bound form of the enzyme.

#### ANM results

We performed ANM calculations for the holoenzyme structure [29] of *Hha*I after removing *in silico* the cofactor. The slowest ANM mode (ANM1) was found to couple the loop motion with small and large domain movements of *Hha*I (Figure 3B–C). The collectivity of this mode is 0.55 [30]. The residue fluctuations driven by ANM1 are compared to the experimental temperature factors in Figure 3D, which indicate close agreement specifically for the loop region, supporting the dominance of this soft mode in the observed fluctuations. This mode exhibits a loop overlap of 0.82 (Table 2). In addition, four other modes among the first ten present a loop overlap above 0.5. The cumulative overlap plot shows that 20 modes (2.3% of all modes) explain 80% of the structural variance (Figure 2A). When we consider 5 modes with high loop overlap, 69% of the variance is explained.

The weighted-average overlap of *Hha*I loop motion based on *p* = 10 softest modes indicates an enhancement factor of 5.2 over random modes (Table 2). The weighted-average overlap as a function of mode index is shown in Figure 3E, obtained by evaluating <O|*_s_*>*_p_* values (Eq. 1) for successive subsets (sliding windows) of *p* = 10 modes along the abscissa. The red arrow indicates the result for the first window composed of ten lowest frequency modes. The bars clearly display the distinctively high loop overlap achieved by the softest modes. The inset displays the overlap difference with respect to random, Δ<O|*_s_*>*_p_*, evaluated for successive segments of length *s* along the backbone sequence. The curve clearly captures the strong correlation at the functional loop (indicated by dashed vertical line).

### Orotidine 5′-phosphate Decarboxylase (OMP decarboxylase)

**Figure 4:**
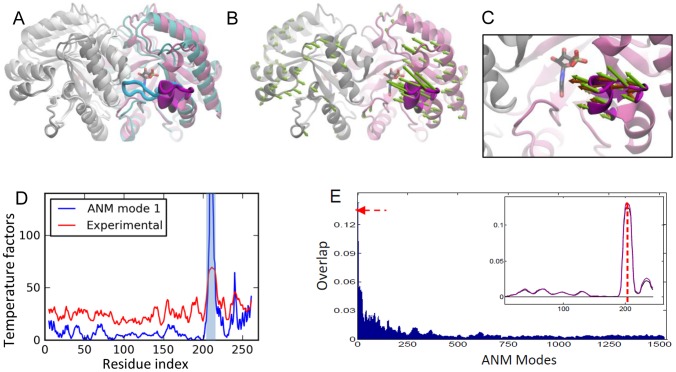
Results for OMP decarboxylase. **A.** Open (*mauve/magenta* loop; PDB id 3GDK [49]) and closed (*cyan/blue loop*; PDB id 3GDL) forms of OMP decarboxylase are shown [49] (in color for one subunit). **B.** ANM mode 1 is shown by *green* arrows. **C.** A close-up view of ANM predicted loop motions compared to experimental changes (*dark orange* arrows). **D.** Theoretical temperature factors calculated using ANM1 compared to experimental B-factors reported for PDB structure 1DQW. Loop region is highlighted in *light blue*. **E.** Same as Figure 3E, for OMP decarboxylase. Curves for A (black) and B (purple) subunits shown in the inset.

Orotidine 5′-phosphate decarboxylase is a homodimeric enzyme with the classic TIM-barrel fold [31]. It catalyzes the conversion of orotidine 5-monophosphate (OMP) to uridine 5-monophosphate (UMP) in the biosynthesis of primidine nucleotides. The active site is located at the dimer interface. A flexible loop located at the C-terminal end is associated with substrate binding and release of the product in the last step of the reaction. The loop (Figure 4A) is in open conformation when it is ordered and in closed conformation at the active site contacting the ligand [31]. Hur and Bruice performed MD simulations and found that the loop changes conformation during the catalytic reaction [32].

#### PCA results

We performed PCA using the seven resolved crystal structures of the enzyme. PC1 and PC2 explain 71% and 26% of the variance, respectively.

#### ANM results

The slowest mode (Figure 4B–C) clearly drives the closure of the loop, exhibiting an overlap of 0.72 with the closure direction (Table 2). The seventh and eighth modes also have high overlap values of 0.79 and 0.7 (not shown). The functional loop is highly mobile in the first mode (Figure 4D). The comparison of residue fluctuations with the experimental temperature factors yields a correlation of 0.68. The cumulative overlap calculations show that 20 modes (1.2% of all modes) explain close to 90% of the structural variance (Figures 2A and S2) and when only those with high loop overlaps (listed in Table 2) are considered 81% of the variance is accounted for.

The weighted-average loop overlap for the 10 slowest modes exhibits a remarkable enhancement (of 11.6) over random modes (Table 2). The weighted-average loop overlap as a function of mode index clearly demonstrates the distinctive overlap achieved by the slowest 10 modes (see the peak indicated in Figure 4E). The overlap difference profile in the inset of Figure 4E also demonstrates how the experimental deformations at the loop regions of both subunits (two curves) correlate with those along soft modes.

### Triosephosphate isomerase (TIM)

**Figure 5:**
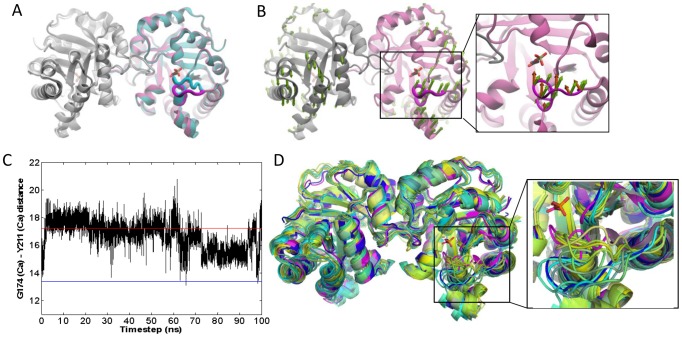
Results for TIM. **A.** Chicken TIM crystal structures in open/apo (*mauve/magenta* loop; PDB id 8TIM) and closed/ligand-bound (*cyan/blue* loop; PDB id 1TPH) forms of the catalytic loop 6 (shown in color for one subunit). **B.** ANM first mode contribution to loop fluctuations. A close-up view of ANM1 induced loop motion (green arrows) in comparison to that experimentally observed (orange arrows). **C.** Change in the distance (Å) between C^α^ atoms of loop residue Gly174 and the relatively immobile residue Tyr211 during 100 ns MD simulation of TcTIM. The distances for closed (*blue* line) and open (*red* line) loop structures are shown as reference. **D.** TcTIM MD snapshots from every 10 ns compared to the closed form (*magenta*), with the inset showing a slightly rotated, enlarged view. Open form is shown in *dark blue*.

The homodimeric enzyme TIM plays a crucial role in the glycolytic pathway by catalyzing the interconversion of dihydroxyacetone phosphate and glyceraldehyde 3-phosphate. Each subunit adopts the TIM-barrel fold as in OMP decarboxylase. Loop 6 that carries a catalytic glutamate closes over the active site and protects it from solvent during catalysis. However, this loop closure is not ligand-gated, i.e. it also takes place in the apo state [17]. Aligned apo and ligand-bound structures of chicken TIM in Figure 5A indicate the conformational change in loop 6 (colored *blue* and *magenta*).

#### PCA results

We performed PCA using 15 resolved crystal structures of the enzyme. PC1 and PC2 explain 52% and 24% of the structural variance, respectively.

#### ANM results

The overall overlap values between PC1 and ANM modes (generated for the apo structure, 8TIM) are lower than 0.4 in general (see Figure S2). In this respect, TIM is one of the four cases (together with biphosphate aldoase, enolase and pyruvate mutase) where the overall structural change between the apo and liganded forms exhibits a relatively weak overlap with ANM soft modes. However, several soft modes contribute to loop motion. Figure 5B displays the loop reorientation driven by mode 1 (with loop overlap of 0.45). ANM calculations repeated with another apo structure, *Trypanosoma cruzi* TIM (TcTIM; PDB identifier 1TCD), corroborated those performed for chicken TIM (Figure S1D).

#### MD results

Our previous 60 ns MD simulation performed for chicken TIM [13] exhibited loop 6 closure, consistent with experimental data. Global deformations that were not apparent in crystal structures have been observed therein to accompany the loop closure. Specifically, the first mode from EDA of the trajectory (with 34% contribution to overall motion) revealed a counter-rotation of the two subunits accompanied by the proper closure of loop 6, again consistent with ANM results. Here we performed an independent MD simulation (100 ns long) on TcTIM apo structure and focused on the distance between the loop and a relatively immobile residue. The results plotted as a function of time (Figure 5C), show multiple opening/closure events in both subunits (only subunit A shown). Snapshots taken every 10 ns indicate various loop conformations between open and closed states (Figure 5D). EDA analysis further confirms that the counter-clockwise rotation of the two subunits in the first mode (with 36% contribution) is coupled to the loop closure event (Figure S1A). The high correlation between the first three modes from EDA and ANM of TcTIM further support the robustness of the results (Figure S1B–C). These findings consistently highlight the coupling between the global counter-rotation of the subunits in the first mode and the loop closure.

### Other enzymes

**Figure 6:**
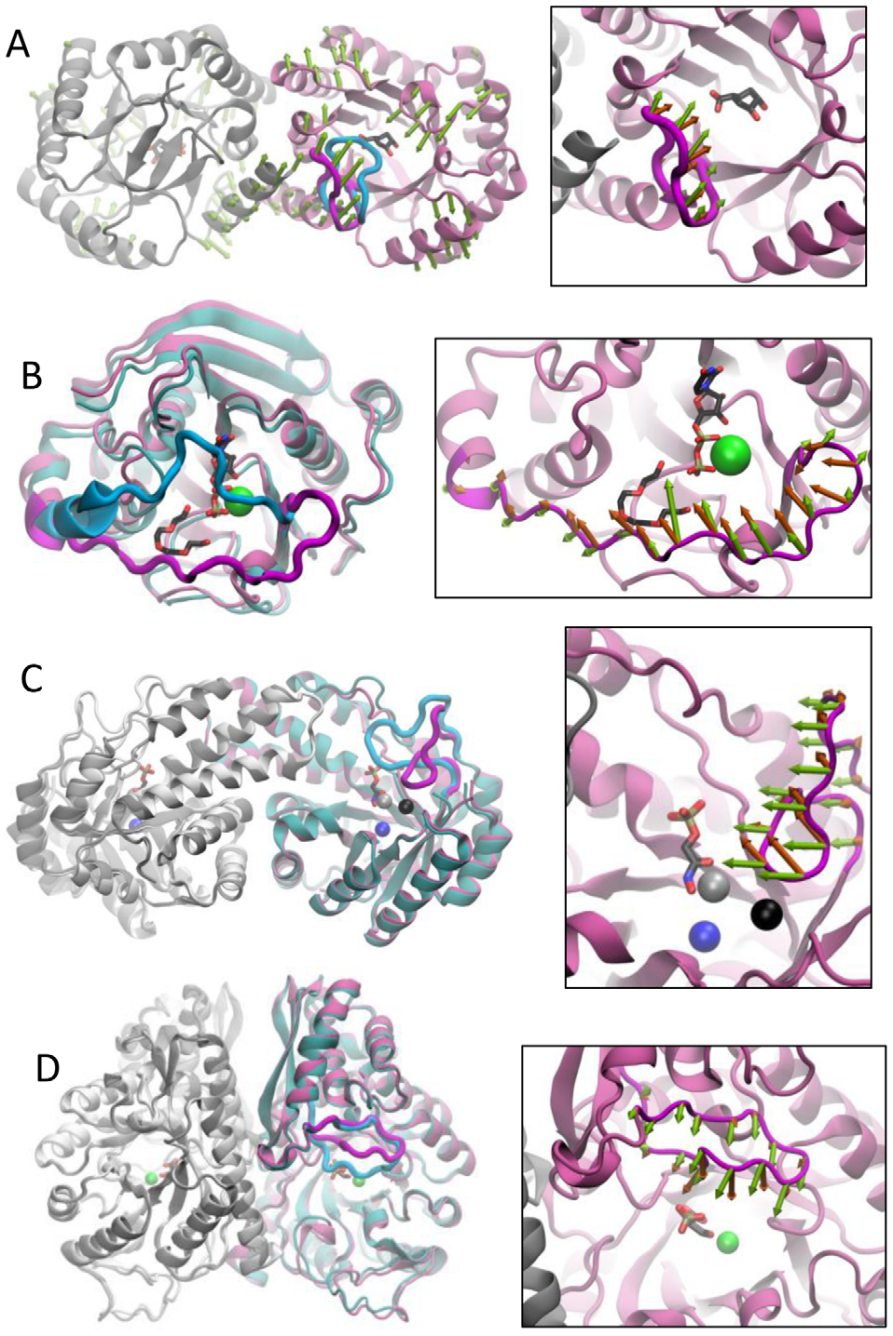
Loop motions from experiments and theory. Enzyme structures are displayed in *mauve* (apo) and *cyan* (liganded), and functional loops in *magenta* (open) and *blue* (closed). In multimeric proteins, ‘other’ chains are colored *grey* and *white*, respectively, for open and closed states. The right panels display the loop regions and compare ANM predictions (*green* arrows) and those experimentally observed (*orange* arrows). Ligands are displayed in stick representation and bound ions are distinguished as spheres. **A.** 3-dehydroquinase apo (1GQN) and liganded (1L9W) structures. Ligand is 3-amino-4,5-dihydrohy-cyclohex-1-enecarboxylate. ANM mode 5 is displayed. **B.** β 1,4-Galactosyltransferase apo (1FGX) and liganded (1NKH) structures, the ligands are uridine-5′-diphosphate and tetraethylene glycol. The *right* panel displays ANM mode 1. **C.** Biphosphate aldoase apo (3C4U) and liganded (3C52). Ligand is phosphoglycolohydroxamic acid. ANM mode 2 is shown. **D.** Enolase apo (3ENL) and bound (7ENL) forms. Ligand is 2-phosphoglyceric acid. ANM mode 3 is displayed.

The results for other proteins are displayed in Figures 6 and S5. Additional data provided in Tables 1 and 2, Figure 2, Tables S1, S2 in Text S1 and Figures S2, S3, S4 essentially consolidate the results described in detail for the four cases.

## Discussion

Proteins undergo a broad range of motions under physiological conditions, spanning from local to global changes in conformations. Among them, the most probable motions, also known as the softest modes, are usually highly collective, i.e., they drive the cooperative motions of entire domains/subunits [10], [33]. Many activities of proteins are achieved, on the other hand, by relatively localized motions, such as loop reconfigurations that accompany ligand binding. A common behavior in all enzymes studied here was the occurrence of the catalytic loop reconfiguration based on the available apo and bound structures. This observation has commonly leaded to the hypothesis that loop motions are triggered by ligand binding.

Given that loop motions are not collective in nature, but seemingly confined to short segments on the backbone, they might be attributed to local, rather than global dynamics. Many studies focused on such ‘regions of interest’ implicitly assuming that the loop reconfiguration observed is predominantly determined by local interactions. Our analysis demonstrates, however, that the local conformational changes observed in experiments at functional loops are not independent of the soft modes of motions intrinsically favored by the architecture. On the contrary, at least in the examined dataset, the soft modes do contribute (more than local high frequency modes) to the reconfiguration of the loops along directions stabilized upon ligand binding.

The top-ranking ANM modes are by definition collective modes of motions known to be highly robust against sequence and structure variations. The correlation between experimentally observed structural changes at the catalytic loops and these modes suggests the evolution of the enzymatic architecture to facilitate the predisposition of the catalytic loop to enzymatic activity. Our previous and current MD simulations on TIM from two different species consistently indicate high mobility and almost full opening/closure of loop 6 in both subunits of the homodimer. In contrast, only half-closure and restricted mobility is observed for the WPD loop during PTP simulations. Evidently, there are other factors that also affect catalytic loop dynamics in terms of reaching the closed state, or the state ‘pre-disposed’ to catalytic activity. One factor may be the favorable electrostatic interactions provided by the substrate (not included in our simulations). Another factor proposed to facilitate loop closure is the presence of the conserved, glycine-rich loops interacting with the active-site loop in previous MD simulations on enolase, β 1,4-galactosyltransferase and lipase [34], [35].

It is important to note that our study does not contradict the critical role of electrostatic interactions in catalysis pointed out earlier [5]–[7], and in fact, our earlier work [36] showed that the catalytic site, once assuming the ‘active’ conformation, is mechanically constrained to maintain its precise geometry required for chemical reactivity. On the other hand, conformational flexibility comes into play, and plays an important role to our view, in facilitating the binding of the substrate, and in favoring the reconfiguration of the active site into its form prone to catalysis, hence the significant role of conformational flexibility in accomplishing catalysis observed in previous work [1]–[4]. In a sense, the structure-encoded flexibility, or the suitable reorientation of the catalytic loop (as shown here to be favored by intrinsic collective motions) is a prerequisite for the ensuing catalytic activity which requires the appropriate chemical (and, in particular, electrostatic) organization.

It is worth noting that the ANM modes are purely based on native contact topology, or geometry. No residue-specific interactions are taken into consideration. The collective dynamics is essentially controlled by uniform spring-like potentials; and these potentials in turn account for the Gaussian fluctuations/distributions of inter-residue distances- the underlying assumption of the theory of elastic networks, as originally set forth for polymer networks [37]. As such, the directions of motions predicted by the ANM are those favored by elastic entropic effects (for a recent review see ref [10]), and the structural changes initiated/favored by these entropic effects are likely to be complemented by enthalpic effects, including in particular electrostatic interactions with the bound ligand to shape and stabilize the final closed conformer. Yet, the he correlation with experimentally observed deformations suggests that these entropic effects play a significant role in defining the accessible mechanisms of ligand binding.

## Methods

### PCA of experimental structural data

The *experimental data* for each protein composed of *N* residues are generated as follows: (*i*) the ensemble of structures is superimposed using an iterative Kabsch algorithm (see SI), (*ii*) mean positions <***R*** *_i_*> = [<*x_i_* > <*y_i_*> <*z_i_*>]^T^ are determined for α-carbons 1≤*i*≤*N* (or those residues with known coordinates), (*iii*) deviations from mean position, Δ***R*** *_i_* ^s^ = [Δ*x*_i_^s^ Δ*y_i_* ^s^ Δ*z_i_* ^s^]^T^ (where Δ*x*_i_^s^ = *x*_i_^s^−<*x_i_*>) are organized in a *3N*-dimensional deformation vector Δ***R*** ^s^ for each structure *s* in the ensemble; (*iv*) the cross-correlations between these deviations, averaged over the entire set are written in a *3N*×*3N* covariance matrix **C** *^(exp)^*, and (*v*) **C** *^(exp)^* is diagonalized to determine the principal modes of structural variations, where 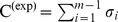 ***p*** *^(i)^* ***p*** *^(i)^* ^**T**^. Here ***p*** *^(i)^* and σ_i_, are the respective *i* ^th^ eigenvector and eigenvalue, and *m* is the number of structures resolved for the studied protein. The principal modes are rank-ordered: PC1 (***p*** *^(1)^*) refers to the direction of maximal variance, succeeded by PC2, etc. The fractional contribution of ***p*** *^(i)^* to structural variance is given by *f_i_* = σ*_i_*/**Σ** *_j_* σ*_j_* where the summation is performed over all modes.

### ANM analysis

The Hessian matrix, **H**, forms the basis of ANM approach. **H** can be written in terms of *N*×*N* submatrices, **H** *^(ij)^*, each of size *3*×*3*, given by
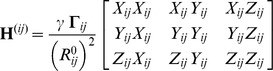
for 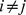, and 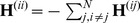. Here 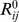 is the magnitude of the distance vector ***R*** *_ij_* ^0^ between α-carbons *i* and *j* (observed in the PDB), and 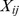, 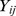, and 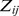 are the components. 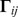 is the *ij^th^* element of the Kirchhoff matrix 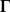 equal to 1 if *i* and *j* are connected (within a cutoff distance of *r_cut_*) in the network, 0 otherwise. A uniform force constant, *γ*, is used for all pairwise interactions. **H** decomposed into *3N-6* nonzero eigenvalues *λ_i_*, and corresponding eigenvectors ***u*** *^(i)^*, as 
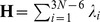
 ***u*** *^(i)^* ***u*** *^(i)^* ^**T**^. ANM covariance is **C**_ANM_ = **H** ^−1^, where **H** ^−1^ is pseudo inverse, such that 1/*λ_1_* is the counterpart of the PCA *σ_1_*, and ***u*** *^(i)^* is the counterpart of ***p*** *^(i)^*.

### Overlap definitions

The *overlap* between PCA and ANM modes is given by the absolute value of the correlation cosine *O_ij_* = |***p*** *^(i)^*. ***u*** *^(j)^*| [9]. For enzymes with two available structures only, ***p*** *^(1)^* is equivalent to the 3*N*-dimensional deformation vector (normalized) between open and closed crystal structures (Table S1 in Text S1). *Cumulative overlap* is defined as 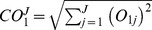 [38]. Note that *CO_1_^J^* = 1 for *J = 3N-6*, i.e., the *3N-6* ANM eigenvectors form a complete set of orthonormal basis vectors.

The orientational correlation between the *s*-residue long loop motions experimentally observed and computationally predicted is measured by the overlap 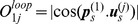 between the loop elements (3*s*-dimensional subvectors) of ***p*** *^(1)^*and ***u*** *^(j)^*. We further define the *weighted-average overlap* for any segment of length *s* based on *p* modes.
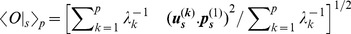
(1)This definition takes account of the magnitudes of loop motions, in addition to their orientations. Calculations were performed using the software package ProDy [39].

### MD and EDA

PTP was simulated for 50 ns in explicit TIP3 [40] water using NAMD [41] with CHARMM force field [42] (see SI for details). Langevin dynamics and Langevin piston Nose-Hoover [43], [44] methods were used to keep the temperature and pressure constant at 300 K and 1 atm. EDA [14] was performed after iterative superposition of the MD trajectory onto the crystal structure. TcTIM simulations were performed using AMBER [45], [46] with the ff03 force field parameters [47], and the protocol described in previous work [13].

## Supporting Information

Figure S1 **Triosephosphate isomerase conformational dynamics.** (**A**) Side view of the first mode of motion obtained by EDA of 100 ns MD trajectory reveals a counter-rotation of the two subunits (blue subunit in front and green subunit at the back) in TcTIM (TIM from *Trypanosoma cruzi)* accompanied by the functional closure of loop 6 (in red). (**B**) Side view of the first ANM mode for TcTIM, also supports the coupling of global deformation and loop closure. (**C**) Overlap matrix for the 10 slowest modes from ANM and EDA. High overlap is observed for the first three modes of ANM and EDA, including the modes shown in panels (A) and (B). (**D**) Overlap matrix for the 10 slowest ANM modes between two different crystal structures of TIM from chicken (8TIM) and parasite TcTIM (1TCD).
(PDF)

Figure S2 **ANM mode overlap with experimentally observed structural change between liganded and unliganded forms of the dataset enzymes.** Overlap of slowest 40 ANM modes, calculated for the whole structure, are shown as bar graph. In addition, the cumulative overlap (see Methods) is displayed as the black curve. Panels are labeled with the name of the proteins.
(PDF)

Figure S3 **Weighted-average overlap of ANM modes with experimentally observed structural change at the loop region.** Weighted-average overlaps (Eq. 1) are calculated using a sliding window of 10 modes starting from slowest modes (*i.e. p* = 1–10, continued as *p* = 2–11, etc.) up to the highest frequency modes (3N-6 of them) for each structure. For the structures with multiple subunits, the results are presented for chain A only. The difference vector between the loop positions from the open to the closed crystal structure, after optimal superimposition of the two structures, is used as the experimental data for loop reconfiguration. The red dashed line indicates the weighted-average overlap value for *p* = 1–10.
(PDF)

Figure S4 **Weighted-average overlap achieved by ten softest ANM modes, relative to that obtained by random modes.** The *difference* Δ<O|*_s_*>*_p_* = <O|*_s_*>*_p_^ANM^*−<O|*_s_*>*_p_^random^* is plotted for loop-sized sliding windows (*s*-residue long segments) along the chain. <O|*_s_*>*_p_^ANM^* is the average over *p* = 10 softest ANM modes (see Eq. 1 in Methods), and <O|*_s_*>*_p_^random^* is computed by generating 10 *random* modes (eigenvectors that obey a Gaussian distribution of residue motions) using the eigenvalues of the original collective modes. The weighted-average overlap value for the functional loop region is marked by red dashed line in each case. Multiple curves correspond to the different subunits in multimeric enzymes. Peaks refer to regions where there is an enhanced difference in overlap with respect to random. Both the size of motions and orientational correlation contribute to weighted average overlaps, hence the need to take the difference with respect to random.
(PDF)

Figure S5 **Loop motions from experiments and theory shown for (A) L-lactate dehydrogenase structures, and (B) Pyruvate mutase.** Same as Figure 6. The PDB identifiers of the structures are: (**A**) 3D0O (apo) and 3D4P (bound); and (**B**) 1S2T (apo) and 1M1B (bound). Ligands are (**A**) nicotinamide-adenine-dinucleotide and pyruvic acid; and (**B**) sulfopyruvate. The enlarged panels display the loop reconfiguration (**A**) between the two structures (*middle*), and the corresponding experimental (orange arrows) and computed (ANM mode 1; green arrows) motions (*right*); and (**B**) predicted by ANM mode 3.
(PDF)

Text S1 **Text S1 gives detailed information about the optimal superimposition of ensembles of structures, MD simulation protocol for PTP and calculation of the covariance matrix.** Text S1 includes Table S1 that lists PDB structure datasets of the enzymes and Table S2 which gives fraction of variance for PCA of overall structure and loop region of the enzymes.
(PDF)
