# Supplementary figures and images for: Coupling between Catalytic Loop Motions and Enzyme Global Dynamics
Source: PLoS Comput Biol. 2012 Sep 27;8(9):e1002705. doi: 10.1371/journal.pcbi.1002705 (PMC3459879; doi:10.1371/journal.pcbi.1002705)

A

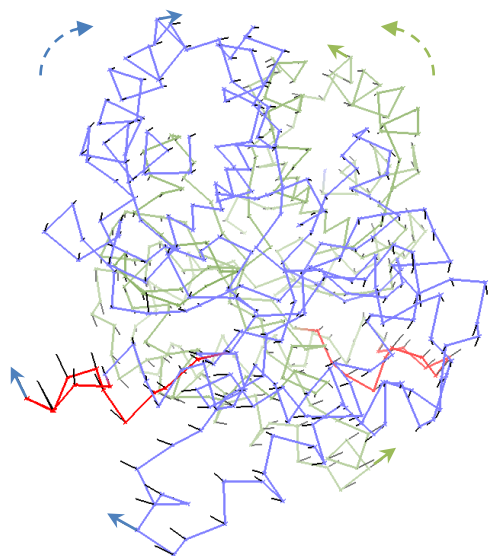

B

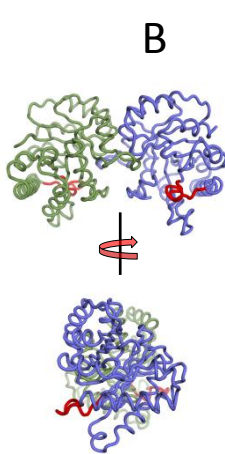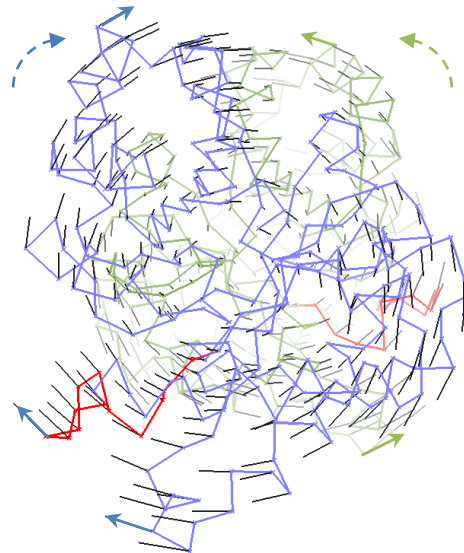

C

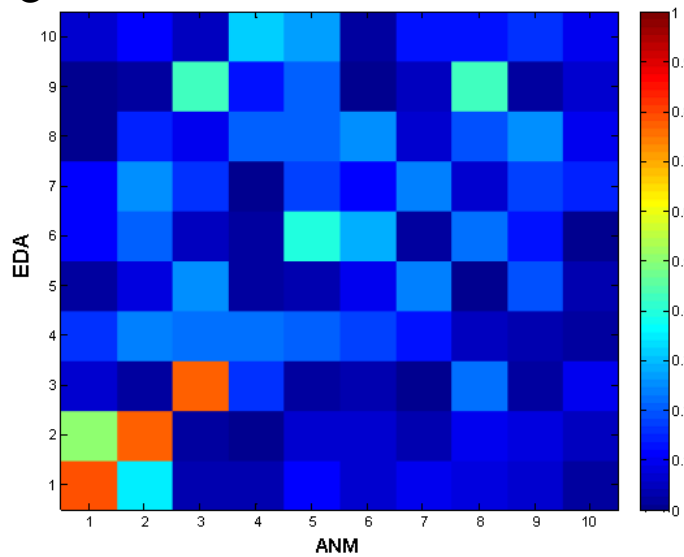

D

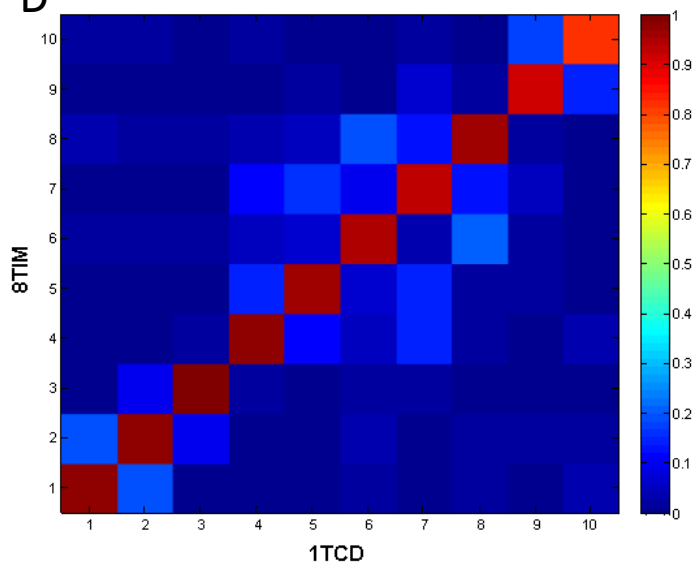

Supplement: Figure S1 — Triosephosphate isomerase conformational dynamics. (A) Side view of the first mode of motion obtained by EDA of 100 ns MD trajectory reveals a counter-rotation of the two subunits (blue subunit in front and green subunit at the back) in TcTIM (TIM from Trypanosoma cruzi) accompanied by the functional closure of loop 6 (in red). (B) Side view of the first ANM mode for TcTIM, also supports the coupling of global deformation and loop closure. (C) Overlap matrix for the 10 slowest modes from ANM and EDA. High overlap is observed for the first three modes of ANM and EDA, including the modes shown in panels (A) and (B). (D) Overlap matrix for the 10 slowest ANM modes between two different crystal structures of TIM from chicken (8TIM) and parasite TcTIM (1TCD). (PDF) [file pcbi.1002705.s001.pdf]

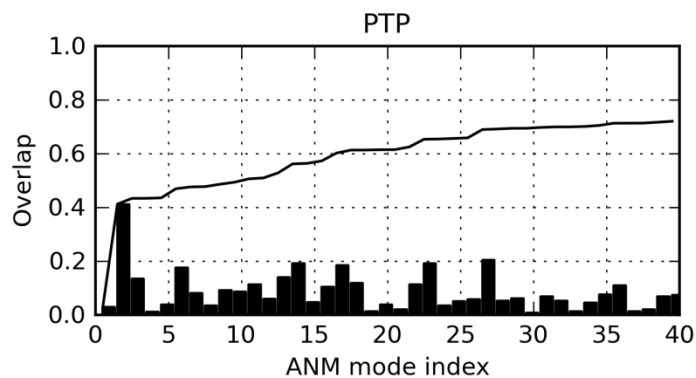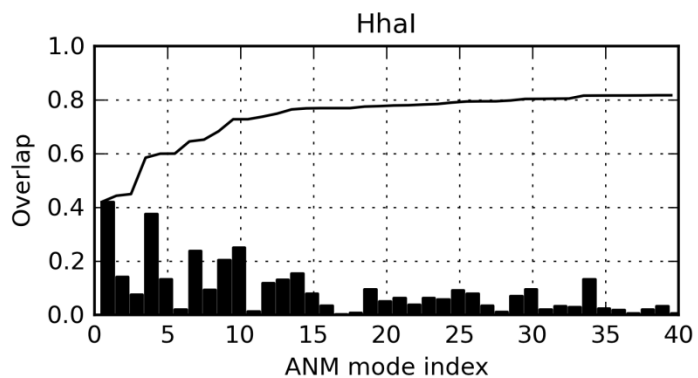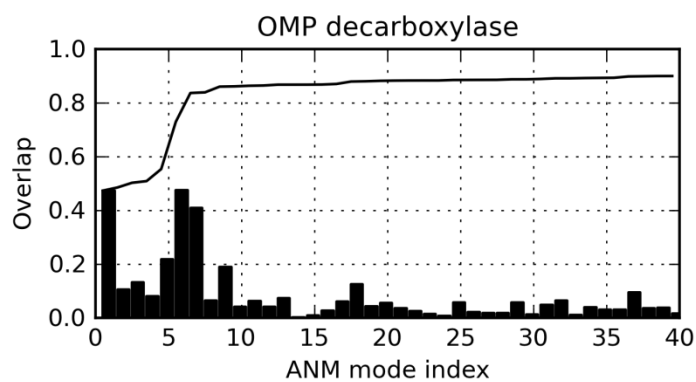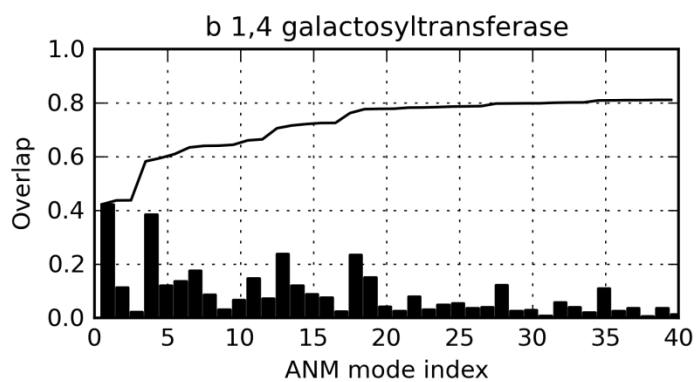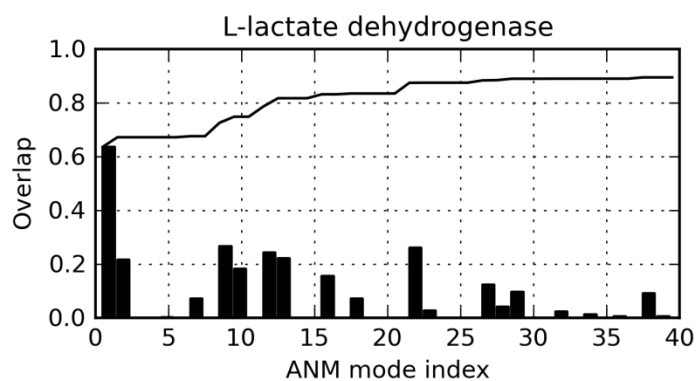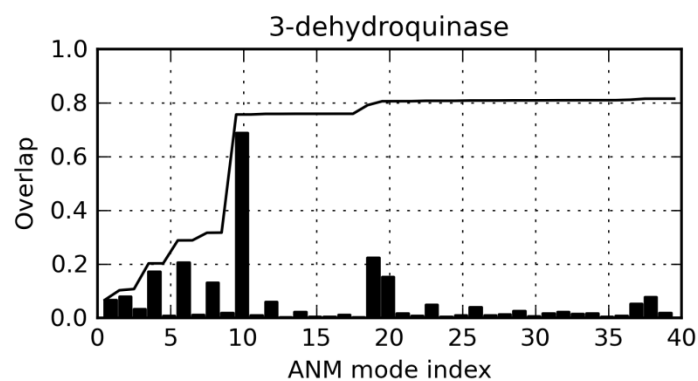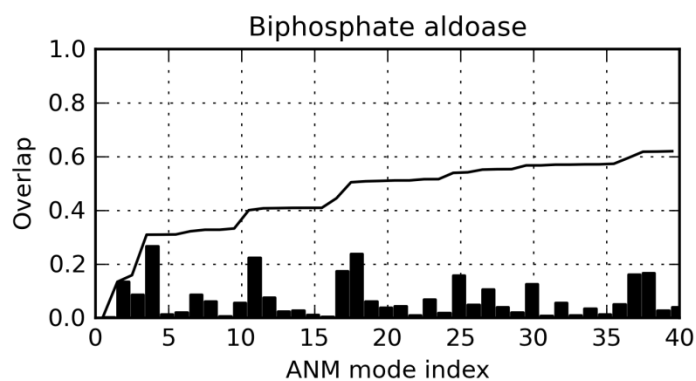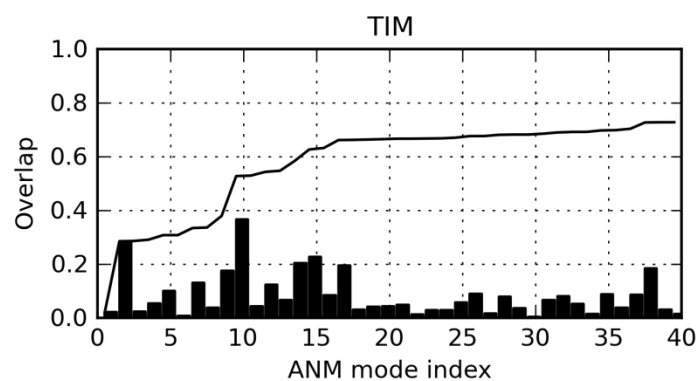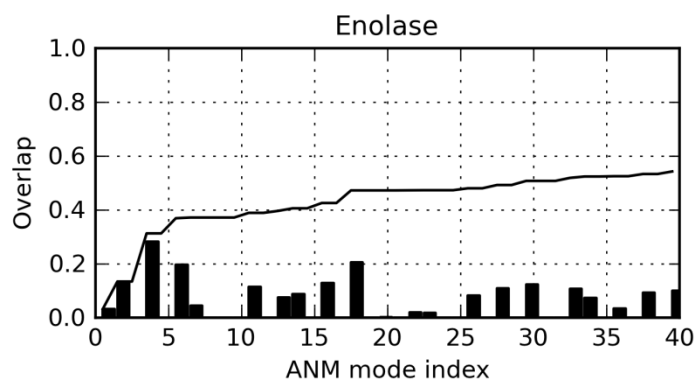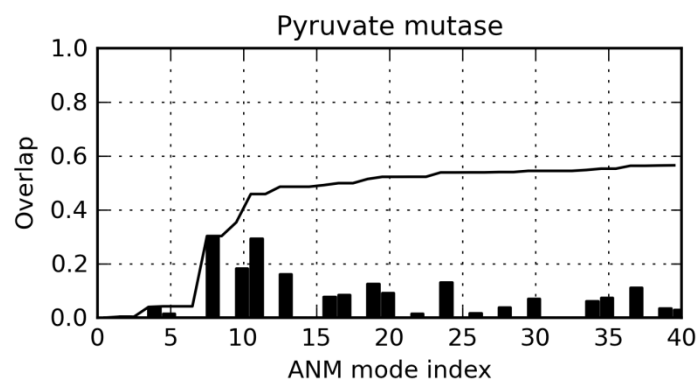

Supplement: Figure S2 — ANM mode overlap with experimentally observed structural change between liganded and unliganded forms of the dataset enzymes. Overlap of slowest 40 ANM modes, calculated for the whole structure, are shown as bar graph. In addition, the cumulative overlap (see Methods) is displayed as the black curve. Panels are labeled with the name of the proteins. (PDF) [file pcbi.1002705.s002.pdf]

A

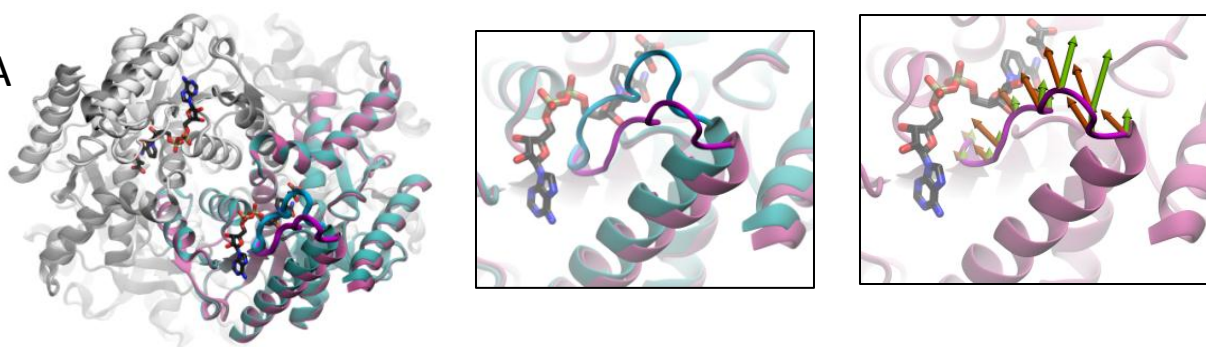

B

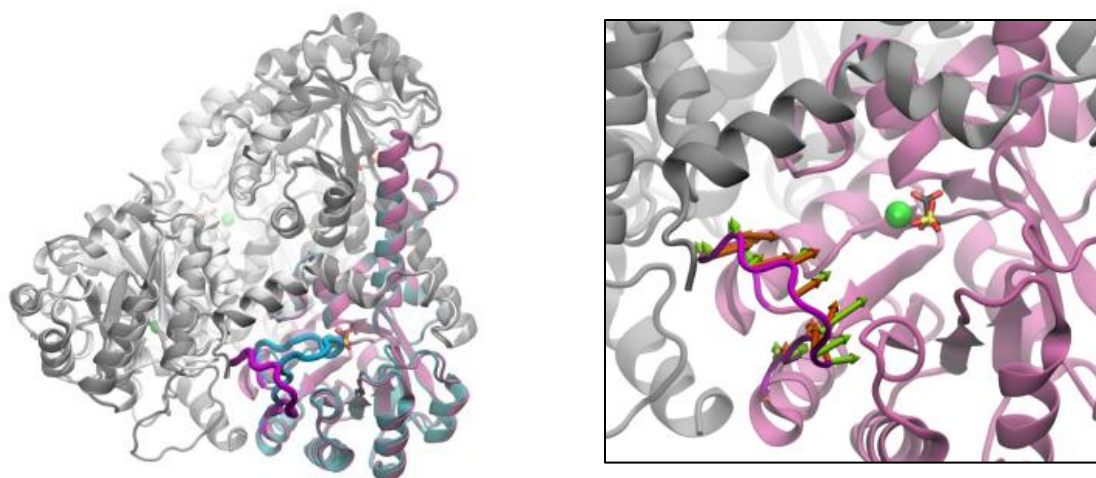

Supplement: Figure S5 — Loop motions from experiments and theory shown for (A) L-lactate dehydrogenase structures, and (B) Pyruvate mutase. Same as Figure 6. The PDB identifiers of the structures are: (A) 3D0O (apo) and 3D4P (bound); and (B) 1S2T (apo) and 1M1B (bound). Ligands are (A) nicotinamide-adenine-dinucleotide and pyruvic acid; and (B) sulfopyruvate. The enlarged panels display the loop reconfiguration (A) between the two structures (middle), and the corresponding experimental (orange arrows) and computed (ANM mode 1; green arrows) motions (right); and (B) predicted by ANM mode 3. (PDF) [file pcbi.1002705.s005.pdf]
