# Supplementary material for: Coupling between Catalytic Loop Motions and Enzyme Global Dynamics
Source: PLoS Comput Biol. 2012 Sep 27;8(9):e1002705. doi: 10.1371/journal.pcbi.1002705 (PMC3459879; doi:10.1371/journal.pcbi.1002705)
Supplement: Figure S3 — Weighted-average overlap of ANM modes with experimentally observed structural change at the loop region. Weighted-average overlaps (Eq. 1) are calculated using a sliding window of 10 modes starting from slowest modes (i.e. p = 1–10, continued as p = 2–11, etc.) up to the highest frequency modes (3N-6 of them) for each structure. For the structures with multiple subunits, the results are presented for chain A only. The difference vector between the loop positions from the open to the closed crystal structure, after optimal superimposition of the two structures, is used as the experimental data for loop reconfiguration. The red dashed line indicates the weighted-average overlap value for p = 1–10. (PDF) [file pcbi.1002705.s003.pdf]

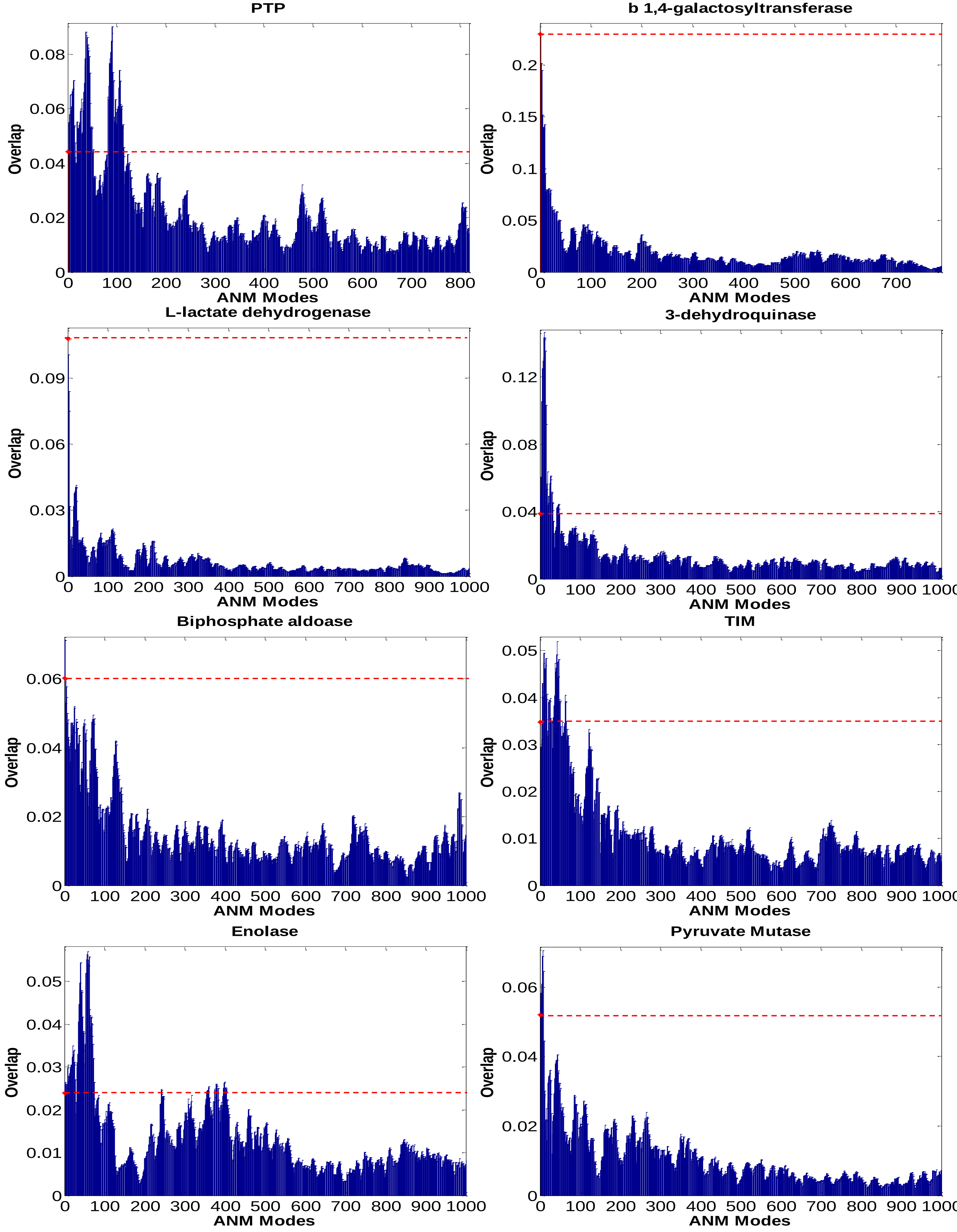
PTP
b 1,4-galactosyltransferase
L-lactate dehydrogenase
3-dehydroquinase
Biphosphate aldoase
TIM
Enolase
Pyruvate Mutase
Overlap
ANM Modes
