# Supplementary material for: Coupling between Catalytic Loop Motions and Enzyme Global Dynamics
Source: PLoS Comput Biol. 2012 Sep 27;8(9):e1002705. doi: 10.1371/journal.pcbi.1002705 (PMC3459879; doi:10.1371/journal.pcbi.1002705)
Supplement: Figure S4 — Weighted-average overlap achieved by ten softest ANM modes, relative to that obtained by random modes. The difference Δ<O|s>p = <O|s>pANM−<O|s>prandom is plotted for loop-sized sliding windows (s-residue long segments) along the chain. <O|s>pANM is the average over p = 10 softest ANM modes (see Eq. 1 in Methods), and <O|s>prandom is computed by generating 10 random modes (eigenvectors that obey a Gaussian distribution of residue motions) using the eigenvalues of the original collective modes. The weighted-average overlap value for the functional loop region is marked by red dashed line in each case. Multiple curves correspond to the different subunits in multimeric enzymes. Peaks refer to regions where there is an enhanced difference in overlap with respect to random. Both the size of motions and orientational correlation contribute to weighted average overlaps, hence the need to take the difference with respect to random. (PDF) [file pcbi.1002705.s004.pdf]

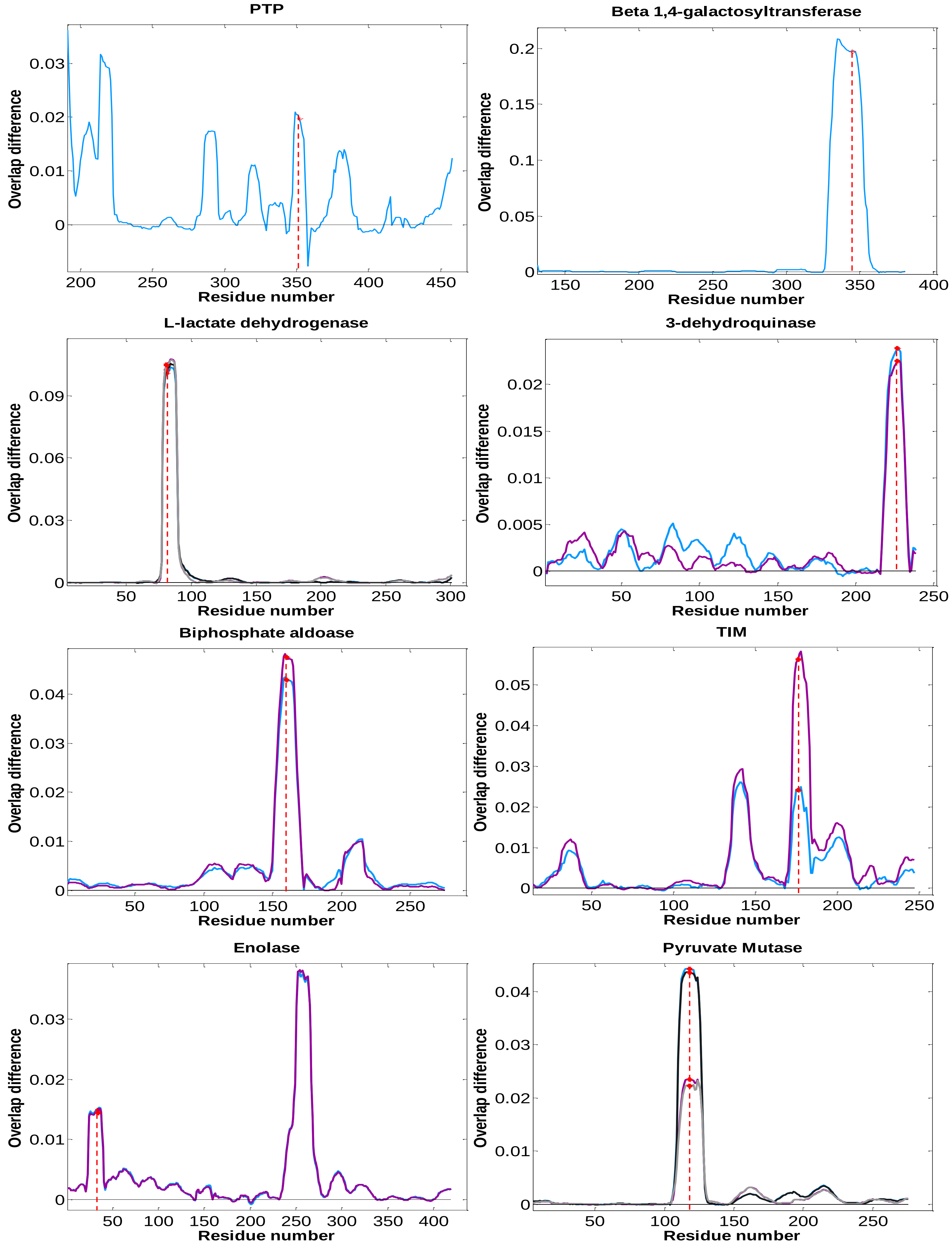
PTP
Overlap difference
0.03
0.02
0.01
0
200 250 300 350 400 450
Residue number
Beta 1,4-galactosyltransferase
Overlap difference
0.2
0.15
0.1
0.05
0
150 200 250 300 350 400
Residue number
L-lactate dehydrogenase
Overlap difference
0.09
0.06
0.03
0
50 100 150 200 250 300
Residue number
3-dehydroquinase
Overlap difference
0.02
0.015
0.01
0.005
0
50 100 150 200 250
Residue number
Biphosphate aldoase
Overlap difference
0.04
0.03
0.02
0.01
0
50 100 150 200 250
Residue number
TIM
Overlap difference
0.05
0.04
0.03
0.02
0.01
0
50 100 150 200 250
Residue number
Enolase
Overlap difference
0.03
0.02
0.01
0
50 100 150 200 250 300 350 400
Residue number
Pyruvate Mutase
Overlap difference
0.04
0.03
0.02
0.01
0
50 100 150 200 250
Residue number
