# Supplementary material for: Coupling between Catalytic Loop Motions and Enzyme Global Dynamics
Source: PLoS Comput Biol. 2012 Sep 27;8(9):e1002705. doi: 10.1371/journal.pcbi.1002705 (PMC3459879; doi:10.1371/journal.pcbi.1002705)
Supplement: Text S1 — gives detailed information about the optimal superimposition of ensembles of structures, MD simulation protocol for PTP and calculation of the covariance matrix. Text S1 includes Table S1 that lists PDB structure datasets of the enzymes and Table S2 which gives fraction of variance for PCA of overall structure and loop region of the enzymes. (PDF) [file pcbi.1002705.s006.pdf]

Iterative procedure for optimal superimposition of ensembles of structures

The procedure consists of the following steps: (i) First, each structure in the ensemble is pairwise superposed onto a randomly selected reference structure using the Kabsch algorithm [1]. (ii) An average set of coordinates is calculated for the superposed set obtained in (i), referred to as the 'average model', (iii) all structures are pairwise superposed on the newly generated 'average model' using the Kabsch algorithm, (iv) steps (ii)-(iii) are repeated until the average model generated in two successive iterations changes by less than the threshold RMSD of 0.001Å. The present superposition method ensures that the structures do not undergo rigid body translational and rotational motions, and allow for direct comparison of the deformation vectors with the ANM eigenvectors describe purely internal motions. This method is used both in PCA of structures and EDA of MD trajectories.

MD simulation details for PTP

System was prepared using PSFGEN, Solvate and AutoIonize plugins of VMD [2]. Solvation box padding distance was set to 6 Å along each direction. Four chloride ions were added to neutralize the system. System was energy minimized for 2000 steps, and equilibrated for 60 ps prior to productive run. Equilibration started at 100 K and the temperature was raised to 300 K in the first 20 ps at increments of 10 K/ps. During equilibration, protein heavy atoms were constrained using harmonic potential with force constant of 0.5 kcal/mol. In equilibration and productive simulation, the cutoff distance was set to 10 Å, all bonds with hydrogen atoms were fixed and integration time step of 2 fs was used.

Calculation of the covariance matrix (from experimental structures, MD snapshots and ANM modes)

The covariance matrix **C** is a $3N$ x $3N$ matrix for a protein of N residues (with known coordinates), which may be written in terms of a set of N x N submatrices $\boldsymbol{C}^{(ij)}$ ($1 \leq i, j \leq$ N), each of size 3 x 3

$$\mathbf{C}^{(ij)} = \begin{bmatrix} \langle \Delta x_i \Delta x_j \rangle & \langle \Delta x_i \Delta y_j \rangle & \langle \Delta x_i \Delta z_j \rangle \\ \langle \Delta y_i \Delta x_j \rangle & \langle \Delta y_i \Delta y_j \rangle & \langle \Delta y_i \Delta z_j \rangle \\ \langle \Delta z_i \Delta x_j \rangle & \langle \Delta z_i \Delta y_j \rangle & \langle \Delta z_i \Delta z_j \rangle \end{bmatrix} \tag{2}$$

Here $\langle \Delta \boldsymbol{x_i} \Delta \boldsymbol{y_j} \rangle$ represents the cross correlation between (i) the X-component of the fluctuation vector $\Delta \boldsymbol{R}_i^s$ representing the departure of the ith residue from its mean position, and (ii) the Y-component of $\Delta \boldsymbol{R}_j^s$ representing the departure of the jth residue from its mean position, averaged over all structures ($1 \le s \le m$) in the examined dataset. The sum of the diagonal elements of $\boldsymbol{C}^{(ij)}$ gives the cross-correlations between the fluctuations of residues i and j as $tr\{\boldsymbol{C}^{(ij)}\} = < \Delta \boldsymbol{R}_i \bullet \Delta \boldsymbol{R}_j >$, and the ith diagonal block gives the mean-square fluctuations of residue i, i.e., $tr\{\boldsymbol{C}^{(ii)}\} = < (\Delta \boldsymbol{R}_i)^2 >$.

Table. S1. PDB structure datasets of the enzymes.

Enzyme	PDB codes					
M.*Hha*I	10mh	1fjx	1hmy	1m0e	1mht	1skm	2c7o
	2c7p	2c7q	2c7r	**2hmy (O)**	2hr1	2i9k	2uyh
	2uyc	2uz4	2z6a	2z6q	2z6u	2zcj	3eeo
	**3mht (C)**	4mht	5mht	6mht	7mht	8mht	9mht
β 1,4-Galactosyltransferase	**1fgx (O)**	1fr8	1nf5	1nhe	**1nkh (C)**	1nmm	1nqi
	1nwg	1o0r	1o23	1oqm	1pzt	1pzy	1tvy
	1tw1	1tw5	1yro	2fyc	2fyd		
L-lactate dehydrogenase	**3d0o (O)**	**3d4p (C)**					
OMP decarboxylase	1dqw	1dqx	**3gdk (O)**	**3gdl (C)**	3gdm	3gdr	3gdt
3-dehydroquinase	**1gqn (O)**	**1l9w (C)**					
Biphosphate aldoase	**3c4u (O)**	**3c52 (C)**					
TIM	1spq	1sq7	1ssd	1ssg	1su5	1sw0	1sw3
	1sw7	1tpb	1tpc	**1tph (C)**	1tpu	1tpv	1tpw
	**8tim (O)**						
PTP	1lyv	1pa9	1qz0	1xxp	1xxv	**1ypt (O)**	1ytn
	**1yts (C)**	1ytw	2i42	3blt	3blu	3bm8	3f99
	3f9a	3f9b					
Enolase	1ebg	1ebh	1els	1l8p	1nel	1one	1p43
	1p48	2al1	2al2	2xgz	2xh0	2xh2	2xh4
	2xh7	2one	**3enl (O)**	4enl	5enl	6enl	**7enl (C)**
Pyruvate mutase	**1m1b (C)**	1pym	**1s2t (O)**	1s2u	1s2v		

Representative open (O) and closed (C) structures used for RMSD and ANM calculations are shown in bold face.

Table S2. Fraction of variance for PCA of overall structure and loop region of enzymes.

	Overall structure			Loop region		
	**PC1**	**PC2**	**PC3**	**PC1**	**PC2**	**PC3**
M.*Hha*I	0.90	0.08	0.01	0.99	0.00	0.00
β 1,4-Galactosyltransferase	0.90	0.06	0.01	0.95	0.04	0.01
OMP decarboxylase	0.71	0.26	0.03	0.87	0.12	0.01
TIM	0.52	0.24	0.11	0.85	0.07	0.03
PTP	0.53	0.15	0.09	0.94	0.02	0.02
Enolase	0.64	0.10	0.07	0.94	0.02	0.01
Pyruvate mutase	0.99	0.02	0.01	1.00	0.00	0.00

Reference List

1. Kabsch W (1976) A solution for the best rotation to relate two sets of vectors. Acta Crystallographica Section A 32: 922-923.
2. Humphrey W, Dalke A, Schulten K (1996) VMD: visual molecular dynamics. J Mol Graph 14: 33-38.
